# National Campaign to Prevent Falls in Construction — United States, 2013

**Published:** 2013-04-26

Each day, on average, two construction workers die in the United States (1). In 2010, the 9.1 million construction workers (including self-employed workers) in the United States accounted for 7% of the national workforce (2), yet experienced 17.1% of fatal work-related injuries (2). In 2011, the rate of fatal injuries in construction was the second highest of any U.S. industry (3). Within the industry, falls at construction sites are the leading cause of death, accounting for 35% of deaths among private sector construction workers (not including government or self-employed workers) in 2011 (1); most of these deaths were attributed to falls from roofs, scaffolds, and ladders (2). Deaths and injuries from falls represent a major, persistent, yet preventable public health problem. Safe construction requires both skilled workers and responsible employers.

CDC’s National Institute for Occupational Safety and Health has engaged the construction sector through a government/labor/management partnership, representing state and federal government agencies, professional organizations, trade associations, labor organizations, and private industry. The goal, in part, is to develop a national campaign aimed at construction contractors, onsite supervisors, and workers to address and reduce falls, fall-related injuries, and fall-related fatalities among construction workers. On Workers’ Memorial Day, April 28, 2013, a national information and media campaign will be launched again through this partnership.
